# Characterization of Japanese cedar bio-oil produced using a bench-scale auger pyrolyzer

**DOI:** 10.1186/s40064-016-1848-7

**Published:** 2016-04-01

**Authors:** Yoshiaki Kato, Ryohei Enomoto, Minami Akazawa, Yasuo Kojima

**Affiliations:** Department of Applied Biochemistry, Faculty of Agriculture, Niigata University, Niigata, 950-2181 Japan; Graduate School of Science and Technology, Niigata University, Niigata, 950-2181 Japan

**Keywords:** Bench-scale auger reactor, Bio-oil, Japanese cedar, Pyrolysis temperature, Secondary reaction, Vapor residence time

## Abstract

A bench-scale auger reactor was designed for use as a laboratory-scale fast pyrolyzer for producing bio-oil from Japanese cedar. An analytical pyrolysis method was performed simultaneously to determine the distribution of pyrolysis products. The pyrolysis temperature was found to have the greatest influence on the bio-oil characteristics; bio-oil yields increased as the pyrolysis temperature increased from 450 to 550 °C. The concentration of levoglucosan in the bio-oil, however, decreased significantly with increasing pyrolysis temperature, while it increased following analytical pyrolysis. The same results were obtained for 4-vinylguaiacol and *E*-isoeugenol, which were the major secondary products produced in the present study. Compared to the yields of these major products obtained via analytical pyrolysis, the yields from the auger reactor were very low, indicating that the auger reactor process had a longer vapor residence time than the analytical pyrolysis process, resulting in the acceleration of secondary reactions of the pyrolysates. The pH values and densities of the bio-oils produced in the auger reactor were similar to those reported by researchers using woody biomass, despite their lower viscosities. From these results, it was concluded that the pyrolysis temperature and residence time of the pyrolysates played a significant role in determining the characteristics of the cedar bio-oil.

## Background

Bio-oil produced via lignocellulosic biomass pyrolysis is considered to be a substitute for fuel oil and diesel in many static applications, such as boilers, furnaces, engines, and turbines for electricity generation and chemical production (Bridgwater 2003). Bio-oil can be produced from wood, bark, agricultural wastes/residues, nuts and seeds, algae, grasses, forest residues, and cellulose and lignin. The differences in the properties of these feedstocks influence the properties of the obtained bio-oils (Mohan et al. 2006). In addition, several important biomass pyrolysis parameters affect bio-oil production: the heating and heat transfer rate, reaction temperature, residence time of the produced vapor, and cooling rate of the vapor (Bridgwater 2003). Reactors used for bio-oil production mainly consist of bubbling fluidized-bed reactors, circulating fluidized bed and transported bed reactors, vacuum pyrolysis reactors, ablative reactors, and auger reactors (Mohan et al. 2006; Bridgwater 2012). In addition, three processes can be used for biomass pyrolysis: slow pyrolysis, intermediate pyrolysis, and fast pyrolysis. They differ based on the residence time of the generated vapor, which leads to different yields of the liquid pyrolysis products contained in the bio-oil (Bridgwater 2012).

Woody biomass is a key potential feedstock for bio-oil production, and most processes have been performed using wood (Ingram et al. 2008). One of the most important plantation species, Japanese cedar (*Cryptomeriajaponica*), accounts for approximately 60 % of the plantation forests in Japan. Currently there is a growing demand to thin these forests (Baba et al. 2011). However, thinned cedar woods are largely left behind as forest residue and not utilized, which leads to reduced thinning activities. Conversely, the thinned woods are available as feedstock for biomass pyrolysis, and therefore their use in bio-oil production may promote additional forest thinning. Japanese cedar pyrolysis has been studied by several researchers. Asmadi et al. (2010) compared the pyrolysis of cedar (softwood) and Japanese beech wood (hardwood) using a closed small ampoule reactor (nitrogen atmosphere, reaction temperature 600 °C, heating time 40–600 s). They described the secondary char formation, char reactivity, tar formation, and subsequent decomposition for both wood species. Chang et al. (2012) pyrolyzed cedar sawdust in a bubbling fluidized-bed reactor in order to evaluate the effects of condensers (with and without spraying) and different fluidizing gases (pyrolysis gas and nitrogen gas) on product yields. They reported that sawdust pyrolysis at 460 °C in pyrolysis gas with spraying in a condenser led to a maximum bio-oil yield.

Of the various pyrolysis reactors used for bio-oil production, auger pyrolysis reactors have a compact design, a lower carrier gas flow, and lower process temperatures (Mohan et al. 2006). Some studies of bio-oil production from woody biomass using auger pyrolysis reactors have been conducted. Ingram et al. (2008) pyrolyzed pine wood, pine bark, oak wood, and oak bark at 450 °C and characterized the physical and chemical properties of the produced bio-oils. Puy et al. (2011) examined the pyrolytic characteristics of pine woodchips from forest residues of two different species at different reaction temperatures, different solid residence times, and different biomass flows, evaluating the process performance, the chemical properties, and the composition of the pyrolysis products. Brown and Brown (2012) pyrolyzed red oak wood and investigated various process parameters, including the heat carrier inlet temperature and mass flow rate, the rotational speed of the screws in the reactor, and the volumetric flow rate of the sweep gas for system optimization. Kim et al. (2014) pyrolyzed pine wood at 500–550 °C using a semi-pilot scale reactor equipped with multistage condensers and characterized the bio-oil generated from each condenser. In these studies, two types of auger reactors were used: single-screw (Ingram et al. 2008; Puy et al. 2011; Kim et al. 2014) and twin-screw (Brown and Brown 2012). The former has a simpler design than the latter, which requires a heat carrier for heat transfer to the feedstock (Brown and Brown 2012). In the present study, a single-screw reactor with a simpler design and no need for a heat carrier was used in the first step of bio-oil production.

Typical fast pyrolysis processes involve short vapor residence times of less than 2 s (Bridgwater 2012). Long vapor residence times cause secondary reactions of the pyrolysis products, which provide products similar to those obtained via fragmentation reaction at high pyrolysis temperatures (Collard and Blin 2014). These fragmentation and secondary reactions of cellulose and hemicellulose during pyrolysis produce low-molecular-weight products, such as formaldehyde, methanol, acetaldehyde, acetone, glycolaldehyde, and acetol (Shen and Gu 2009; Asmadi et al. 2010; Shen et al. 2010; Branca et al. 2013). Lignin pyrolysis at high temperatures affords phenols and catechols via secondary reactions (Asmadi et al. 2010). These reactions can also increase the yield of non-condensable gases, such as CO, CO_2_, H_2_, and CH_4_ (Shen and Gu 2009; Asmadi et al. 2010; Patwardhan et al. 2011). Enhanced CO formation during cellulose and hemicellulose pyrolysis at elevated temperatures has been reported (Shen and Gu 2009; Shen et al. 2010; Qu et al. 2011). Conversely, the formation of CO_2_ decreased at elevated temperatures during cellulose pyrolysis (Qu et al. 2011). The release rates for CH_4_ and H_2_ were observed to reach a maximum at 500–600 °C and ~600 °C, respectively, during lignocellulose pyrolysis (Yang et al. 2007). Furthermore, the gas yield from wood pyrolysis was observed to increase at longer vapor residence times (Asmadi et al. 2010). The formation of CO was also promoted at longer vapor residence times during cellulose pyrolysis (Shen and Gu 2009). The suppression of secondary reactions is also important for bio-oil production via fast pyrolysis (Mohan et al. 2006).

The objective of the present study was to evaluate the effects of pyrolysis temperature on the properties of bio-oil obtained from Japanese cedar. In addition, the pyrolysis reactions for cedar bio-oil production using a bench-scale auger reactor were compared to those of an analytical fast pyrolysis process.

## Results and discussion

### Properties of the Japanese cedar feedstock

The properties of the Japanese cedar wood meal prepared from a whole trunk were established using proximate and ultimate analyses. These results are shown in Table 1. Compared to Scots pine and Black pine (Puy et al. 2011), the cedar had lower volatile matter and higher fixed carbon and carbon contents. The value for fixed carbon relates to char yield from biomass pyrolysis.

**Table 1:** Proximate and ultimate analysis of Japanese cedar woodchip prepared from whole trunk

Proximate analysis (wt%)	
Moisture	15.5
Volatile matter	66.6
Ash	0.3
Fixed carbon^a^	17.6
Ultimate analysis (wt%)	
C	50.0
H	6.1
O^a^	43.9

^a^By difference

The results of the ultimate analysis performed in the present study are similar to those for Japanese cedar wood meal reported by Phuphuakrat et al. (2010).

### Effect of pyrolysis temperature on the yields of char, gas, and bio-oil

**Fig. 1:**
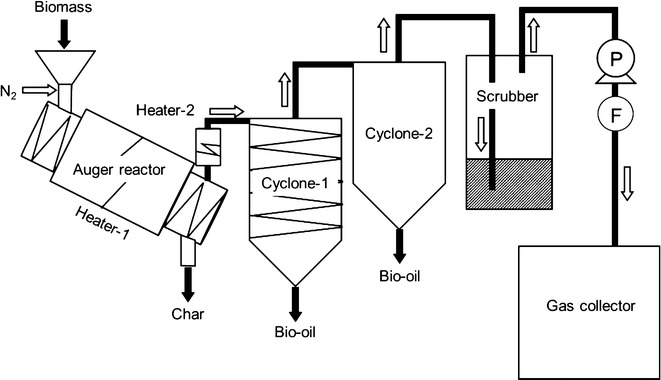
Bench-scale auger reactor for bio-oil production. *P* pump, *F* flow meter

Bio-oil production from cedar wood meal using the bench-scale auger reactor was performed at pyrolysis temperatures ranging from 450 to 550 °C, as shown in Fig. 1. The effects of the reaction temperature on the yields of char, gas, and bio-oil are shown in Table 2. The bio-oil yield increased significantly with increasing pyrolysis temperature from 450 to 500 °C, but only slightly from 500 to 550 °C. These results suggested that, using the auger reactor, the maximum cedar bio-oil yield could be obtained at around 550 °C. The change in gas yield with temperature was similar to that of the bio-oil yield, whereas the char yield followed an opposite trend. According to Bridgwater (2012), the yield of pyrolysis products in bio-oil reaches a maximum at reaction temperatures between 480 and 520 °C depending on the feedstock, and the maximum yield of bio-oil obtained via fast pyrolysis is 75 wt%. In addition, bio-oil production from pine wood using auger reactors has been investigated by several researchers. Ingram et al. (2008) obtained bio-oil yields ranging from 48.7 to 55.2 wt% at 450 °C, while Puy et al. (2011) and Kim et al. (2014) obtained bio-oil yields of 58.7 and 59.8 wt% at 500 °C, respectively. Thus, the cedar bio-oil yields obtained in the present study are comparable to bio-oil yields obtained previously using auger reactors. The changes in the yields of gas and char obtained from cedar wood meal as a function of the pyrolysis temperature are also similar to previously reported yields (Puy et al. 2011; Bridgwater 2012; Kim et al. 2014).

**Table 2:** Yield of char, gas, and bio-oil produced from cedar using auger reactor at various pyrolysis temperatures

Pyrolysis temperature (°C)	Yield (wt% of dry feedstock)		
	Char	Gas^a^	Bio-oil^b^
450	35.4	10.7	53.9
500	22.2	16.9	60.9
550	21.3	17.2	61.4

^a^By calculation from gas analysis data at 25 °C

^b^By difference

Cedar bio-oils produced in the auger reactor were collected directly from the bottoms (without ethanol washing) of two cyclones cooled at different temperatures (see Fig. 1). After each cedar bio-oil production run, greater than 90 % of the total bio-oil yield was obtained from Cyclone-1. Therefore, the bio-oils collected from the bottom of Cyclone-1 were used to determine their physical and chemical properties. Kim et al. (2014) produced pine bio-oil using an auger reactor combined with three condensers; the first and second condensers were equipped with heat exchangers, and third condenser was cooled to ~25 °C. They reported that the total bio-oil yield from the three condensers reached 59.8 wt% of the feedstock.

### Effect of pyrolysis temperature on the physical properties of the bio-oil

The physical properties of the cedar bio-oils collected from Cyclone-1 are presented in Table 3. The moisture contents of the bio-oils produced at 450–550 °C ranged from 49.8 to 58.1 wt%, which was higher than the moisture content of typical bio-oils produced via fast pyrolysis (Mohan et al. 2006) and pine bio-oil produced using a semi-pilot auger reactor (Kim et al. 2014). The vapor residence time in the auger reactor used in the present study for cedar bio-oil production was longer than that in a typical fast pyrolysis process (Mohan et al. 2006), which can lead to further thermal decomposition of pyrolysates in the vapor phase via secondary reactions. In addition, the molecular weight distribution in the cedar bio-oil was lower than that in bio-oil produced by fast pyrolysis (see below). Furthermore, the feedstock for the cedar bio-oil production had a higher moisture content than that for pine bio-oil production at pyrolysis temperatures ranging from 500 to 550 °C (Kim et al. 2014). The moisture content of the pine bio-oil ranged from 37 to 39 wt%, which was higher than that for typical bio-oils. Therefore, it can be concluded that the high moisture content of the produced cedar bio-oil resulted from the high feedstock moisture content and long vapor residence time. Kim et al. (2014) also suggested that water in bio-oil may arise not only from the moisture naturally present in the feedstock, but also dehydration and cross-linking reactions of cellulose and hemicellulose during pyrolysis and certain operating conditions, such as longer solid and vapor residence times.

**Table 3:** Physical properties of cedar bio-oils produced at various pyrolysis temperatures

Pyrolysis temperature^a^ (°C)	Density (g/ml)	pH	Moisture content (wt%)	Viscosity (mPa s)
450	1.13	2.00	49.8	5.77
500	1.15	2.16	58.1	4.53
550	1.13	2.06	52.1	3.79

^a^Bio-oils were collected from the bottom of Cyclone-1

The physical characteristics of typical wood bio-oils (at 40 °C and 25 % water) are as follows: density, 1.20 kg/L; pH, 2.5; and viscosity, 40–100 mPa s (Bridgwater 2003). The cedar bio-oils collected in the present study had density and pH values similar to those for typical bio-oils, but lower viscosities, which were influenced by their higher moisture contents. This result is in agreement with those of Lou et al. (2004), who produced bio-oils from four different types of plant biomass and found that bio-oil with high moisture, which was produced from rice straw, contents had lower kinetic viscosity values.

The pyrolysis products in bio-oil that are derived from lignin consist of oligomers and have detrimental effects on the properties of bio-oil, such as its viscosity, reactivity, and stability (Bayerbach and Meier 2009). The water-insoluble faction of bio-oil mainly consists of pyrolysis products derived from lignin and is referred to as pyrolytic lignin (Scholze et al. 2001; Mohan et al. 2006; Bayerbach and Meier 2009). Thus, in order to determine the molecular weight distributions of pyrolytic lignin (Scholze et al. 2001) in the bio-oils produced from cedar, the molecular weight distributions of the cedar bio-oils produced at various temperatures were determined using GPC analysis at a frequency of 280 nm, and the results are listed in Table 4. The weight-average molecular weights (Mw) of the bio-oils ranged from 334 to 399, which was similar to that of the water-insoluble factions (Mw: 328–556) of pine bio-oils produced using an auger reactor (Kim et al. 2014). However, the Mw of the cedar bio-oils were less than that of the pyrolytic lignin obtained from softwood bio-oil produced via fast pyrolysis (Scholze et al. 2001). Compared to softwood bio-oil production via fast pyrolysis, cedar bio-oil production in the present study using an auger reactor involved a longer residence time in the vapor phase, which can cause secondary reactions of the pyrolysis products. These secondary reactions can result in the production of smaller molecules. Therefore, secondary reactions that occur during the longer vapor residence time may influence the production of pyrolysates from the cedar lignin in the cedar bio-oil. Kim et al. (2014) also suggested that the first and second cracking of vapors using longer solid and vapor residence times cause the production of smaller pyrolysis products. Conversely, the number-average molecular weights (Mn) of the cedar bio-oils obtained in the present study ranged from 114 to 124, which was smaller than that for the lignin-rich fraction of the pine bio-oil reported in the literature (Kim et al. 2014). GPC analysis at 280 nm can detect phenol, furfural, and 5-hydroxymethylfurfural, which are all smaller than guaiacol and were detected in the cedar bio-oils produced in the present study (see below). Mn values are strongly affected by the presence of small molecules in mixtures of molecules with different molecular weights. These results indicate, therefore, that small molecules present in the cedar bio-oil, such as furans and phenol, resulted in a decreased Mn value.

**Table 4:** Molecular weight distribution of cedar bio-oils produced at various pyrolysis temperatures

Pyrolysis temperature^a^ (°C)	Mn	Mw	Mw/Mn
450	124	375	3.02
500	114	334	2.94
550	118	399	3.39

^a^Bio-oils were collected from the bottom of Cyclone-1

### Identification of the pyrolysis products in the cedar bio-oil

**Fig. 2:**
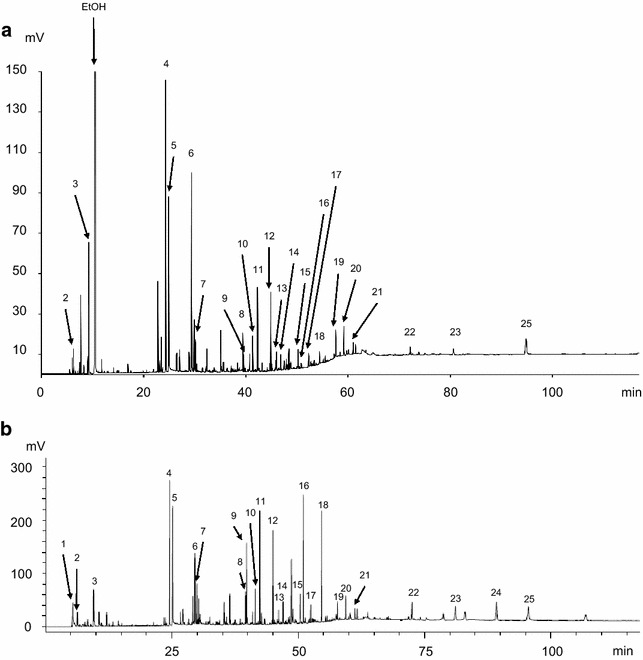
Chromatograms of **a** cedar bio-oil produced at 500 °C and **b** analytical cedar pyrolysis at 500 °C

Rather than the absolute area, the area percentage served as the dependent variable in order to eliminate any inconsistencies due to the variations in sample size and product carryover. It was confirmed that the contribution of the area for a given peak was statistically similar between experiments (Akazawa et al. 2015). A typical chromatogram for the cedar bio-oil is shown in Fig. 2a, and the major pyrolysis product assignments are listed in Table 5. The major pyrolysis products obtained from analytical cedar pyrolysis, for which a typical chromatogram is shown in Fig. 2b, are also listed in Table 5. Peak no. 1 moved through the GC column at a speed similar to the carrier gas velocity and was not detected in the liquid phase, bio-oil. Analytical pyrolysis enables the detection of non-condensable gas and condensable vapors, which are directly analyzed after pyrolysis. Therefore, it was indicated that it is a gaseous product. Lignocellulose pyrolysis mainly produces gases such as methane, hydrogen, carbon monoxide, and carbon dioxide (Qu et al. 2011). Moreover, hydrocarbon gases such as methane, ethane, and ethylene are detectable using GC/FID (Holm 1999). These results indicated that peak no. 1 was likely methane. The listed major pyrolysis products were classified into eleven groups as follows: methane (peak no. 1), acetaldehyde (peak no. 2), methanol (peak no. 3), acetol (peak no. 4), glycolaldehyde (peak no. 5), acetic acid (peak no. 6), furans (peak nos. 7, 8, and 19), cyclic ketones (CK, peak nos. 9 and 10), guaiacyl compounds (peak nos. 11, 12, 14–18, and 20–24), phenol (peak no. 13), and levoglucosan (peak no. 25). The major pyrolysis products derived from lignocellulose are cellulose (Hosoya et al. 2007; Patwardhan et al. 2009; Shen and Gu 2009), hemicellulose (Hosoya et al. 2007; Branca et al. 2013), and lignin (Hosoya et al. 2007; Asmadi et al. 2010), and it has been previously reported that their pyrolysis products, except methane, are present in bio-oils (Milne et al. 1997).

**Table 5:** Major pyrolysis products identified in chromatograms of cedar bio-oil produced at 500 °C and analytical cedar pyrolysis at 500 °C

Peak no.	Compounds	Area %	
		Bio-oil	Analytical pyrolysis
1	Methane	N.D.	1.90
2	Acetaldehyde	0.69	1.15
3	Methanol	5.30	1.99
4	Acetol	14.13	7.18
5	Glycolaldehyde	8.44	6.73
6	Acetic acid	12.05	4.80
7	Furfural	1.48	1.08
8	2(5H)Furanone	1.69	1.40
9	2-Hydroxy-2-cyclopenten-1-one	0.97	3.98
10	2-Hydroxy-3-methyl-2-cyclopenten-1-one	1.58	1.59
11	Guaiacol	3.85	5.11
12	4-Methylguaiacol	3.60	3.26
13	Phenol	0.88	0.54
14	4-Ethylguaiacol	0.74	1.03
15	Eugenol	1.14	1.56
16	4-Vinylguaiacol	0.26	6.06
17	*Z*-Isoeugenol	0.98	1.12
18	*E*-Isoeugenol	0.61	5.18
19	5-Hydroxymethylfurfural	1.26	0.82
20	Vanillin	1.77	1.39
21	Acetoguaiacone	0.70	0.63
22	Dihydroconiferylalcohol	0.67	1.68
23	Coniferylaldehyde	0.67	2.28
24	Coniferylalcohol	N.D.	2.96
25	Levoglucosan	2.81	3.00

### Effect of pyrolysis temperature on the gas composition

The non-condensable gas produced during cedar pyrolysis using the auger reactor was collected and its composition was analyzed via GC/TCD. The results are shown in Table 6 and compared to the yield of methane obtained for the analytical cedar pyrolysis process. When the pyrolysis temperature was raised from 450 to 550 °C, the concentrations of H_2_, CH_4_, and CO increased, while that of CO_2_ decreased. During analytical cedar pyrolysis, the yield of CH_4_ also increased when the pyrolysis temperature was raised from 450 to 550 °C. Puy et al. (2011) reported increases in the concentrations of H_2_, CH_4_, and CO and a decrease in that for CO_2_ when pine woodchips were pyrolyzed using an auger reactor at pyrolysis temperatures of 500 to 600 °C. These results indicate that the generated yields vary differently as a function of the pyrolysis temperature. As mentioned above, the gas yield from lignocellulose pyrolysis is affected by the pyrolysis temperature and vapor residence time, which influence the primary and secondary reactions, respectively. Compared to a typical fast pyrolysis process for bio-oil production (Mohan et al. 2006), the auger reactor for cedar bio-oil production had a longer residence time in the vapor phase. Therefore, the gas formed during cedar bio-oil production may be due to both primary and secondary reactions.

**Table 6:** Chemical compositions of gases yielded by cedar pyrolysis at various pyrolysis temperatures

Pyrolysis with auger reactor (°C)	Gas composition (vol%)^a^				Area %	
	H_2_	CO	CH_4_	CO_2_	Analytical pyrolysis (°C)	CH_4_
450	0.89	43.44	1.27	54.40	450	1.9
500	1.98	45.36	7.74	44.92	500	8.4
550	4.30	47.79	13.03	34.88	550	16.4

^a^By calculation from gas analysis data

### Effect of pyrolysis temperature on the chemical properties of the bio-oil

**Fig. 3:**
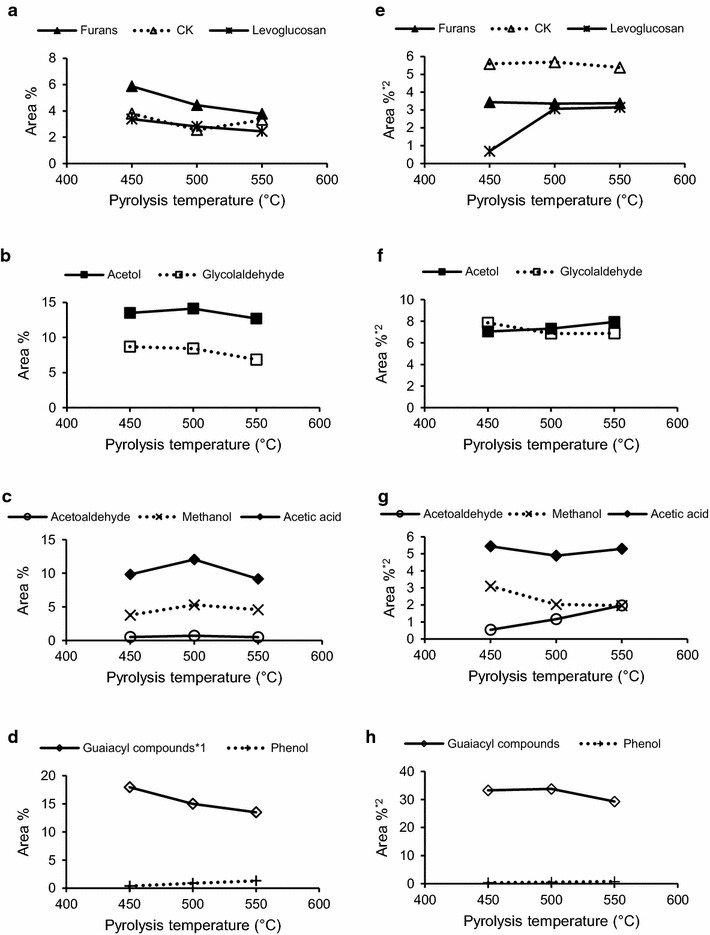
Yields of cedar pyrolysis products at various pyrolysis temperatures. **a**–**d** Cedar bio-oil analysis, **e**–**h** analytical cedar pyrolysis. Furans: peak nos. 7, 8, and 19; Cyclic ketones (CK): peak nos. 9 and 10; Guaiacyl compounds: peak nos. 11, 12, 14–18, and 20–24. *^1^Except for peak no. 24, *^2^Calculated using the total peak area without peak no. 1

The pyrolysis temperature clearly influences the product distribution in a bio-oil. In the present study, the effect of the pyrolysis temperature on the product distribution in cedar bio-oils produced using the bench-scale auger reactor shown in Fig. 1 was investigated. The results are shown in Fig. 3a–d and compared to those for analytical cedar pyrolysis (Fig. 3e–h). In Fig. 3e–h, the peak area percentages were calculated from the total peak area without peak no. 1 (methane), which is not detected in liquid phase, bio-oil. When the pyrolysis temperature was increased from 450 to 550 °C, the total peak area percentage of the major pyrolysis products in the bio-oil decreased from 67.6 to 58.1 %. In particular, the temperature increase from 500 to 550 °C resulted in a decreased total area percentage from 66.3 to 58.1 %. For the analytical pyrolysis, the total area percentage of the major pyrolysates without peak no. 1 (methane) also decreased from 67.4 to 60.5 % when the pyrolysis temperature increased from 500 to 550 °C, but barely changed when the temperature was raised from 450 to 500 °C. These results indicate that high pyrolysis temperatures above 500 °C lead to an increased production of other pyrolysates.

An increased pyrolysis temperature from 450 to 550 °C decreased the area percentages for the major pyrolysis products in the bio-oil, except for acetaldehyde, methanol, acetol, acetic acid, and phenol (Fig. 3a–d). The concentrations of the first four products increased when the temperature increased from 450 to 500 °C (Fig. 3c), while that of phenol increased when the temperature was raised from 450 to 550 °C (Fig. 3d). These pyrolysates are produced via fragmentation and/or secondary reactions (Shen and Gu 2009; Asmadi et al. 2010; Branca et al. 2013). It should be noted that the effect of the pyrolysis temperature on the yields of pyrolysates was different for the analytical cedar pyrolysis and cedar bio-oil production processes. The yield of levoglucosan increased during analytical cedar pyrolysis when the temperature was raised from 450 to 550 °C (Fig. 3e). Furthermore, the yields of acetol and acetic acid were lower following analytical pyrolysis (Fig. 3f, g) than were observed in the cedar bio-oil (Fig. 3b, c). The auger reactor had a longer vapor residence time than that of the micro-pyrolyzer used for analytical pyrolysis, which may have promoted secondary reactions of the pyrolysates in the vapor phase in the former. Notably, levoglucosan produced via cellulose pyrolysis is thermally decomposed in the vapor phase by secondary reactions (Hosoya et al. 2008; Ronsse et al. 2012). These results indicate that the different effects of the pyrolysis temperature on the yields of pyrolysates were due to fragmentation and secondary reactions. Conversely, the guaiacol yields in the cedar bio-oil were lower than those for analytical cedar pyrolysis (Fig. 3d, h), which may be affected by the collectability of the bio-oil from the auger reactor because the bio-oil has to be analyzed after the collection from condensers while in analytical pyrolysis, pyrolysates can be directly analyzed without the collection.

**Fig. 4:**
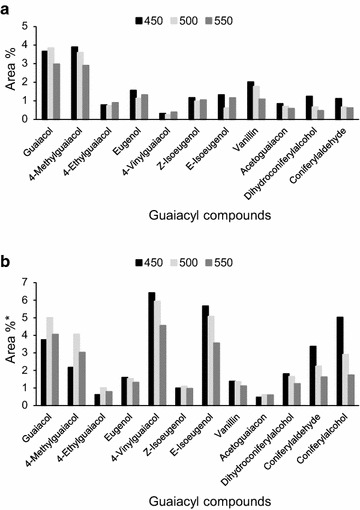
Yields of guaiacyl compounds generated during cedar pyrolysis at various temperatures. **a** Cedar bio-oil analysis, **b** analytical cedar pyrolysis. *Calculated using the total peak area without peak no. 1

Regardless of the differences in the reactor systems, however, the overall effect of the pyrolysis temperature on the yield of guaiacyl compounds was similar for analytical cedar pyrolysis and cedar bio-oil production (Fig. 3d, h, respectively). The differences in the systems still did influence each guaiacol yield. The temperature effect of each pyrolyzer on the yield of each guaiacyl compounds was evaluated, as shown in Fig. 4. Coniferyl alcohol was undetectable in the cedar bio-oil produced with the auger reactor (Fig. 4a), and its yield decreased with increasing pyrolysis temperature from 450 to 550 °C during analytical cedar pyrolysis (Fig. 4b). Coniferyl alcohol is an intermediate in the formation of coniferyl aldehyde and dihydroconiferyl alcohol via pyrolysis; and these two products were detected in the cedar bio-oil (Fig. 4a). Pathways for the formation of these products have been proposed as described below (Akazawa et al. 2015). Coniferyl aldehyde is formed following the elimination of two proton radicals from the γ-hydroxymethyl group of the side chain of coniferyl alcohol. This reaction is likely initiated by the homolytic cleavage of the γ-hydroxyl group. Dihydroconiferyl alcohol is obtained via the addition of two proton radicals to the C_α_–C_β_ unsaturated bond in coniferyl alcohol, which is also involved in the formation of 4-ethyl guaiacol from 4-vinyl guaiacol. Coniferyl alcohol is also an intermediate for the formation of other guaiacyl compounds, such as vanillin, *E*-isoeugenol, 4-vinyl guaiacol, 4-methyl guaiacol, and guaiacol. *E*-Isoeugenol is an intermediate for the formation of eugenol, 4-vinyl guaiacol, and *Z*-isoeugenol. Coniferyl alcohol pyrolysis was previously conducted at 400–600 °C using Py-GC/MS, and its degradation was accelerated at temperatures above 500 °C. The auger reactor used in the present study for cedar bio-oil production had a longer vapor residence time than the micro-pyrolyzer used for analytical pyrolysis. These results indicate that the thermal degradation of coniferyl alcohol is more sensitive to long vapor residence times than pyrolysis temperatures ranging from 450 to 550 °C. In addition, the yields of 4-vinyl guaiacol and *E*-isoeugenol in the cedar bio-oil (Fig. 4a) were particularly low compared to those observed for analytical cedar pyrolysis (Fig. 4b), which indicates that the longer vapor residence time in the auger reactor also affected the formation of these pyrolysates. Therefore, it can be concluded that secondary reactions of lignin pyrolysates are affected by both high pyrolysis temperatures and long vapor residence times.

## Conclusions

Japanese cedar pyrolysis at 450–550 °C using a bench-scale auger reactor was conducted to determine the effects of pyrolysis temperature on bio-oil production. The obtained cedar bio-oil had a lower viscosity than typical bio-oils, likely due to its high moisture content and low molecular weight distribution. In the cedar bio-oil, the yield of levoglucosan (derived from cellulose) decreased at elevated temperatures, while it increased following analytical cedar pyrolysis. Furthermore, the yields of 4-vinylguaiacol and *E*-isoeugenol derived from lignin were lower in the bio-oil than in the pyrolysates obtained following analytical pyrolysis. The auger reactor used for cedar bio-oil production had a longer vapor residence time than the micro-pyrolyzer used for the analytical pyrolysis, which likely promoted secondary reactions of the pyrolysates in the vapor phase. Therefore, it can be concluded that the properties of the cedar bio-oil were influenced by both the pyrolysis temperature and the vapor residence time.

## Methods

### Material

Woodchips prepared from a Japanese cedar (*C. japonica*) trunk were provided by Azuma–Sansho Co., Ltd, Akita, Japan. After air-drying, the woodchips were milled using a hammer mill with a 3-mm screen (Sansho Industry Co., Ltd). The milled chips were sieved to particle sizes below 0.71 mm (analytical fast pyrolysis) and from 0.71 to 2 mm (bio-oil production). The properties of the sieved cedar feedstock were determined as follows: the proximate analysis was performed according to Japanese industrial standard methods and the ultimate analysis was provided by Shimadzu techno-research.

### Analytical pyrolysis

Analytical pyrolysis of cedar wood meal was performed using two pyrolysis–gas chromatography (Py-GC) systems: a Py-GC/flame ionization detector (Py-GC/FID) consisting of a micro-pyrolyzer (EGA/PY-3030D, Frontier lab) and a GC-2010 Plus (Shimadzu), and a Py-GC/mass spectrometer (Py-GC/MS) consisting of a micro-pyrolyzer (JCI-22, JAi) and a JMS T-100GCV GC-TOFMS instrument (JEOL). Samples (0.5 mg) were pyrolyzed at 450, 500, 550, and 600 °C using the Py-GC/FID and at 590 °C using the Py-GC/MS. For both Py-GC analyses, the GC was equipped with an Rtx-Wax capillary column (60 m × 0.25 mm i.d.; 0.25 µm film thickness, RESTEK). The injector temperature was maintained at 250 °C, and split injection was used with a 1:100 split rate. The column oven temperature was held at 40 °C for 5 min and then raised to 250 °C at a rate of 4 °C/min. The temperature was then maintained for 60 min. For the Py-GC/MS analysis, a NIST mass spectral library was employed to identify each peak. After identification, each peak of the chromatograms resulting from Py-GC/FID analysis was identified based on the retention times of authentic samples (methane (GL Science), methanol, acetic acid, guaiacol, phenol, eugenol, 5-hydroxymethylfurfural, coniferylalcohol, levoglucosan (Wako), 4-methylguaiacol, furfural (TCI), and 2(5H)furanone (Aldrich)) and the results obtained via Py-GC/MS analysis.

### Bio-oil production using a bench-scale auger reactor

Bio-oils were produced from cedar wood meal at 450, 480, 500, and 550 °C using the bench-scale auger reactor shown in Fig. 1. The wood meal was fed at 5.23–6.35 g/min for 90 min along with 2 L/min nitrogen into the cylindrical reactor (inside diameter: 40 mm, length: 789 mm) with an internal auger lying along the reactor’s axis. The distance between the screws of the auger was approximately 28 mm, and the auger speed was 20 rpm. The heating zone for pyrolysis was 305 mm long along the reactor axis. In the heating zone, N_2_ velocity calculated from the reactor inside diameter and N_2_ flow is more than 2.65 cm/s, which indicates that the vapor residence time in that zone can be shorter than 11.5 s. After pyrolysis, the char was moved to a vapor-char separation zone and dropped into a char collector. The vapor-char separation zone was lower than the chip supply zone in the reactor, which was inclined at an angle of 11°. To separate the non-condensable gases and bio-oil, two cyclones (Cyclone-1 and Cyclone-2) were cooled with two water cooling circulation systems at 20 and 4 °C, respectively. The non-condensable gases were washed with a wet scrubber and collected in gas bags at 5 L/min. The collected gas was analyzed using a GC/thermal conductivity detector (TCD) GC-7000 (J-science lab) as previously reported (Kojima et al. 2014). Ultimate analysis of the char was provided by Shimadzu techno-research.

### Determination of the physical and chemical properties of the generated bio-oils

The water content of the obtained bio-oils was determined via Karl Fischer titration. The pH of the samples was measured using a HM-30V pH meter (Toa) at room temperature. Dynamic viscosities of the bio-oils were determined using a VM-10A-L viscometer (Sekonic) at 40 °C.

The product distributions of the bio-oils were analyzed via GC/MS and GC/FID. Prior to each analysis, the bio-oil was diluted to approximately 60–70 wt% with ethanol. The analytical conditions were the same as those described above for the analytical pyrolysis.

The molecular distributions of the bio-oils were determined using gel permeation chromatography (GPC). GPC was performed using an LC-2000 HPLC system (JASCO) with a photodiode array (PDA) detector (frequency from 200 to 400 nm). GPC KF-802 and KF-801 columns (Shodex) were used with tetrahydrofuran (THF) containing 10 mM phosphate as the mobile phase flowing at 0.6 ml/min. Samples for GPC were prepared by dissolving the bio-oil in THF at approximately 10 wt%. Each bio-oil solution in THF was filtered using a 0.45-μm filter prior to GPC analysis. The GPC column was standardized using polystyrene molecular weight standards in the range from 108 to 197,000 g/mol. Therefore, the molecular weights of the bio-oils as determined by GPC were polystyrene equivalent molecular weights.

## Abbreviations

*C. japonica*: *Cryptomeria**japonica*
CK: cyclic ketones
FID: flame ionization detector
GPC: gel permeation chromatography
Mn: number-average molecular weights
MS: mass spectrometer
Mw: weight-average molecular weights
PDA: photodiode array
Py-GC: pyrolysis-gas chromatography
TCD: thermal conductivity detector
THF: tetrahydrofuran
